# Monitoring the hematologic markers in patients undergoing single-stage exchange arthroplasty for periprosthetic joint infection

**DOI:** 10.1186/s42836-025-00330-1

**Published:** 2025-09-23

**Authors:** Wenbo Mu, Juan D. Lizcano, Boyong Xu, Wentao Guo, Abudousaimi Aimaiti, Xiaogang Zhang, Javad Parvizi, Li Cao

**Affiliations:** 1 Department of Orthopaedics, First Affiliated Hospital of Xinjiang Medical University, Urumqi, 830054 Xinjiang China; 2 Rothman Orthopaedic Institute at Thomas Jefferson University, Philadelphia, PA 19107 USA; 3 International Joint Center, Acibadem University Hospital, Istanbul, 34303 Turkey; 4 Ministry of Education, Key Laboratory of High Incidence Disease Research in Xingjiang (Xinjiang Medical University, Urumqi, Xinjiang 830054 China; 5 Xinjiang Clinical Research Center for Orthopedics, Urumqi, Xinjiang 830054 China

**Keywords:** Periprosthetic joint infection, Single-stage exchange arthroplasty, Hematologic parameters

## Abstract

**Background:**

Periprosthetic joint infection (PJI) is a serious complication that necessitates a complex treatment strategy. Single-stage exchange arthroplasty, combined with intravenous and intra-articular antibiotic infusions, has shown high efficacy in treating complex PJIs. However, the impact of this approach on hematologic parameters remains underexplored. This study aims to evaluate the postoperative trends in blood platelet count, white blood cell (WBC) count, and neutrophil count in patients undergoing single-stage exchange arthroplasty.

**Methods:**

A retrospective analysis was conducted on 313 patients who underwent single-stage revision for PJI between June 2010 and October 2022. Hematologic parameters were monitored for the first seven postoperative days. The delta between preoperative and lowest postoperative values for platelet, WBC, and neutrophil counts was calculated. Statistical analyses compared these changes between revision total hip arthroplasty (rTHA) and revision total knee arthroplasty (rTKA) groups.

**Results:**

Platelet count significantly decreased postoperatively, reaching its nadir on day 2.5 for rTHA and day 2.8 for rTKA. The delta in platelet count was higher in rTHA patients (73.5 × 10^9^/L) compared to rTKA patients (46.0 × 10^9^/L). The incidence of thrombocytopenia was higher in the rTHA group (28.7%) compared to the rTKA group (12.3%). Multivariate regression analysis identified rTHA and preoperative platelet levels as independent risk factors for greater postoperative platelet decreases. WBC and neutrophil counts initially increased postoperatively, peaking on day 1, and then gradually declined, with nadirs around day 4–5.

**Conclusion:**

Single-stage revision for PJI is associated with significant postoperative decreases in platelet count, particularly in patients undergoing rTHA. However, this hematologic change did not result in bleeding complications and may not represent a major clinical concern in most patients. Routine monitoring remains advisable to guide perioperative management.

## Background

Periprosthetic joint infection (PJI) is a challenging complication in orthopedic surgery, associated with significant psychosocial stress, morbidity, and healthcare costs [1–3]. The severity and complexity of PJI necessitate effective management strategies to combat these infections and preserve joint function. Single-stage exchange arthroplasty, combined with intravenous and intra-articular antibiotic infusions, has proven effective in managing complex PJI [4–9].

While the efficacy of this approach is well-documented, the overall impact of single-stage exchange arthroplasty for PJI, combined with prolonged antibiotic use, on hematological parameters has not been thoroughly investigated [10]. Previous studies on total hip arthroplasty (THA) have noted that inflammatory blood cells, such as white blood cells (WBCs) and neutrophils, increase in the first 24 h after surgery, indicating an inflammatory response provoked by the surgery [11]. Research in various medical fields has shown that fluctuations in platelet, WBC, and neutrophil count can be significantly impacted by extended antibiotic therapy. These changes can have severe consequences, such as increased risk of opportunistic infections and postoperative bleeding, complicating the patient's recovery process [12–16]. Moreover, studies suggest that the incidence of these hematological abnormalities is higher than commonly anticipated, underscoring the need for regular monitoring of blood parameters in patients undergoing prolonged antibiotic therapy [12, 17–19]. However, the specific trends and impacts of these hematological changes in the context of PJI treatment have not been reported.

Our study aims to describe the trends in postoperative platelet count, WBC count, and neutrophil count among PJI patients treated with a combination of intravenous and intra-articular antibiotic infusions in single-stage revisions. We aim to identify the time points at which these hematological parameters reach their nadir and determine the factors associated with significant variation in these blood counts.

## Materials and methods

Following the approval by the institutional ethics committee (K202404-44), our study retrospectively analyzed patient records at a tertiary hospital from June 1, 2010, to October 30, 2022. This analysis targeted patients who received single-stage exchange arthroplasty with concurrent intra-articular and intravenous antibiotic infusions for PJI, which was diagnosed based on the 2018 International Consensus Meeting criteria [20]. The decision to proceed with a single-stage approach was based on institutional protocols. At our institution, single-stage revision is routinely performed under broad indications, including both culture-positive and culture-negative PJI cases, provided that patients exhibit favorable local and systemic conditions, such as a good soft tissue envelope, absence of active systemic sepsis, and no severe immunocompromise. This practice has previously demonstrated satisfactory infection control outcomes when combined with intra-articular antibiotic infusion strategies [8, 10]. Patients with bilateral PJI, multi-joint PJI, fungal infections, or cases requiring amputation after PJI treatment were excluded from the analysis.

### Patient population

The cohort included 313 patients undergoing treatment for PJI, with an average age at index surgery of 64.1 ± 11.3 years (revision total hip arthroplasty (rTHA): 61.4 ± 14 years; revision total knee arthroplasty (rTKA): 67.5 ± 9.5 years, *P* < 0.001) and a mean body mass index (BMI) of 24.7 ± 3.9 kg/m^2^ (revision hip arthroplasty: 24.5 ± 4.1 kg/m^2^; revision knee arthroplasty: 25.8 ± 3.7 kg/m^2^, *P* = 0.002). The cohort comprised 59.1% females (revision hip arthroplasty: 50.3%; revision knee arthroplasty: 67.9%, *P* = 0.001) (Table 1).

**Table 1:** Baseline demographics

Variables	rTHA (*n* = 151)	rTKA (*n* = 162)	*P* value
Age (y)	61.4 ± 14.0	67.5 ± 9.5	< 0.001
BMI (kg/m^2^)	24.5 ± 4.1	25.8 ± 3.7	0.002
Sex			0.001
Female (%)	76 (50.3)	110 (67.9)	
Male (%)	75 (49.7)	52 (32.1)	
CCI	2.4 ± 1.7	2.6 ± 1.1	0.199
Culture results			0.529
Positive (%)	108 (71.5)	122 (75.3)	
Negative (%)	43 (28.5)	40 (24.7)	
IV antibiotic type			0.026
Vancomycin (%)	126 (83.4)	148 (91.4)	
Carbapenem (%)	22 (14.6)	14 (8.6)	
Vancomycin + Carbapenem (%)	3 (2.0)	0 (0.0)	
IV antibiotic duration (d)	13.6 ± 2.7	13.3 ± 1.6	0.327
Topical antibiotic infusion type			0.078
Vancomycin (%)	83 (55.0)	108 (66.7)	
Carbapenem (%)	22 (14.5)	14 (8.6)	
Vancomycin + Carbapenem (%)	46 (30.5)	40 (24.7)	
Topical antibiotic duration (d)	13.2 ± 2.3	13.6 ± 1.6	0.111
WBC preop (× 10^9^/L)	6.8 ± 2.1	6.7 ± 1.7	0.783
WBC mean lowest value (× 10^9^/L)	6.7 ± 2.2	6.7 ± 2.0	0.878
WBC mean day of lowest values (d)	4.4 ± 1.4	4.2 ± 1.7	0.324
Delta WBC from preop to lowest value (× 10^9^/L)	0.18 [− 12.1:6.6]	0 [− 0.9:6.4]	0.211
Leukopenia (*n*, %)	7 (4.7)	1 (0.6)	0.020
Neutrophil preop (× 10^9^/L)	4.3 ± 1.8	4.3 ± 1.5	0.835
Neutrophil mean lowest value (× 10^9^/L)	4.4 ± 1.9	4.5 ± 1.9	0.975
Neutrophil mean day of lowest values (d)	4.6 ± 1.3	4.3 ± 1.7	0.104
Delta neutrophil from preop to lowest value (× 10^9^/L)	− 0.15 [− 11.1:6.3]	− 0.24 [− 10.1:7.2]	0.225
Neutropenia (*n*, %)	4 (2.6)	0 (0.0)	0.017
Platelet preop (× 10^9^/L)	281.2 ± 99.2	283.9 ± 101.2	0.819
Platelet mean lowest value (× 10^9^/L)	193.7 ± 73.1	231.1 ± 80.0	< 0.001
Platelet mean day of lowest values (d)	2.5 ± 1.2	2.8 ± 1.7	0.449
Delta Platelet from preop to lowest value (× 10^9^/L)	73.5 [− 229:368]	46.0 [− 358:257]	< 0.001
Thrombocytopenia (*n*, %)	43 (28.7)	19 (12.3)	< 0.001
Transfusion (*n*, %)	78 (51.7)	27 (16.7)	< 0.001

*BMI* body mass index, *CCI* Charlson comorbidity index, *IV* intravenous, *WBC* white blood cell, *preop* pre-operative

### Surgical technique

A tourniquet was used throughout the case in all knee revisions. For rTKA, a midline incision with a medial parapatellar approach was used, whereas rTHA was approached through a posterolateral incision. Initial steps involve the excision of any existing skin scars and sinus tracts to reduce infection sources. Intraoperative collection of joint fluid facilitates the identification of causative microorganisms through culture analysis. The debridement process focuses on the removal of all infected, necrotic bone and tissue to establish a clean surgical margin characterized by viable, bleeding soft tissue. This is followed by chemical debridement, executed through sequential lavages with saline, povidone-iodine, and hydrogen peroxide, to further ensure an environment hostile to microbial survival.

Adopting a dual surgical setup, the procedure involves a comprehensive re-sterilization phase mid-surgery. This includes the removal and replacement of used drapes, gowns, gloves, surgical sets, suction catheters, electrocautery, and light handles. The surgical team then regowns with new sterile attire and new instruments for the re-implantation phase is brought in. A second lavage of the surgical field is performed using pulsatile lavage to effectively remove any residual micro-debris and membrane from the acetabular cavity, femoral, and tibial canals. Knee revisions predominantly use cemented fixation, while hip revisions are approached with a preference for cementless fixation.

Depending on culture results, 0.5 g of vancomycin or carbapenems (meropenem or imipenem) powder was applied intraoperatively into the femoral and tibial canals in knee revisions and into the femoral canal and acetabulum in hip revisions prior to prosthesis implantation. Postoperatively, intra-articular antibiotic infusion commenced on the first day after surgery. For culture-negative cases, patients received intravenous vancomycin (1 g every 12 h), along with alternating intra-articular injections of vancomycin (0.5 g in the morning) and a carbapenem (0.5 g in the afternoon). Each dose was diluted in 10 mL of sterile normal saline and injected under aseptic conditions. For culture-positive cases, both intravenous and intra-articular antibiotics were tailored to the identified pathogens based on antimicrobial susceptibility testing, as determined by a Multidisciplinary infectious disease team. Prior to each intra-articular injection, synovial fluid was aspirated using a T-branch connector in hip surgeries or syringe aspiration in knee procedures. Surgical drainage was clamped before each injection and reopened 3 h before the subsequent dose to optimize intra-articular drug retention. This regimen was continued for 10–16 days. Subsequently, patients transitioned to an oral regimen of Quinolones and Rifampin until erythrocyte sedimentation rate (ESR) and C-reactive protein (CRP) levels normalized, for a minimum of 31 days [10, 21].

Prior to 2018, patients undergoing revision surgery were routinely given 40 mg of low-molecular-weight heparin once daily and oral rivaroxaban for ten days as part of their venous thromboembolism (VTE) prophylaxis, unless there was ongoing postoperative bleeding. Since 2018, our protocol has gradually shifted to using only 40 mg of low-molecular-weight heparin once daily as the primary VTE prevention method. All patients started ankle pump exercises and hip and knee bending on the first day after surgery to encourage mobilization [22]. All patients received intravenous tranexamic acid at a dose of 20 mg/kg/h during surgery, with additional local application of tranexamic acid as needed during the surgery, and 1 g of tranexamic acid was applied locally in the joint cavity before closure. Postoperative care included routine blood tests for the first seven postoperative days to monitor recovery and detect any adverse hematological effects of antibiotic therapy. Normal ranges for hematological parameters were defined based on our laboratory standards as follows: a WBC count within 3.5–9.5 × 10^9^/L, a neutrophil count within 1.8–6.3 × 10^9^/L, and a platelet count within 125–350 × 10^9^/L. Values falling below these specified ranges are classified as leukopenia, neutropenia, and thrombocytopenia, respectively. To assess the changes in hematological parameters, we calculated the difference or delta between preoperative values and the lowest postoperative measurement for WBC count, neutrophil count, and platelet count. This approach allowed us to quantify the extent of hematological changes over time. We also documented demographic details, significant comorbidities, Charlson comorbidity index (CCI) scores, and the incidence of blood transfusions during hospitalization.

### Data analysis

All statistical analyses were performed using JMP statistical software (version 17.2.0; JMP Statistical Discovery, Cary, NC, USA). Continuous variables were presented as mean ± standard deviation (SD) or median with interquartile range (IQR), depending on the normality of the data distribution. Categorical variables were expressed as frequencies and percentages. Normality of the data was assessed using the Shapiro–Wilk test. An independent t-test was used for normally distributed data, and the Mann–Whitney U test was used for non-normally distributed data. Categorical variables were compared using the chi-square test or Fisher’s exact test, as appropriate. Demographic and clinical data were compared between rTHA and rTKA. The mean day of the lowest recorded value for WBC, neutrophil count, and platelet count was compared between the rTHA and rTKA groups using independent t-tests. A multivariate regression model was used to identify risk factors for an increase in the delta in platelet counts from preoperative to the lowest postoperative values. Statistical significance was set at *P* < 0.05 for all tests.

## Results

### Platelet count

**Fig. 1:**
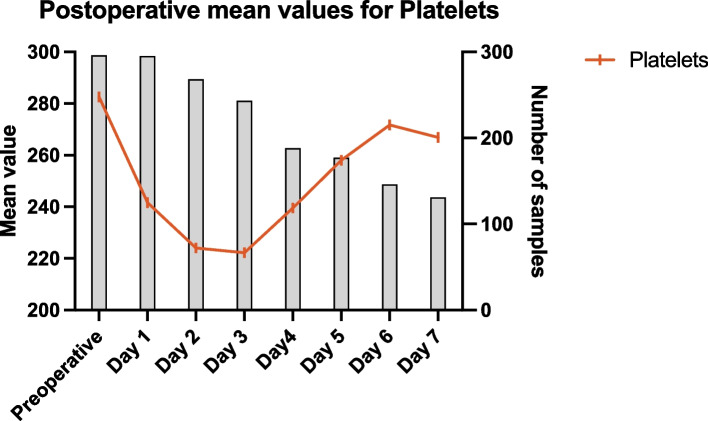
Postoperative mean values for Platelets: Mean platelet counts are plotted from preoperative day to postoperative days 1 through 7, demonstrating the trend and changes in platelet levels over time. The number of samples taken each day is also indicated

**Fig. 2:**
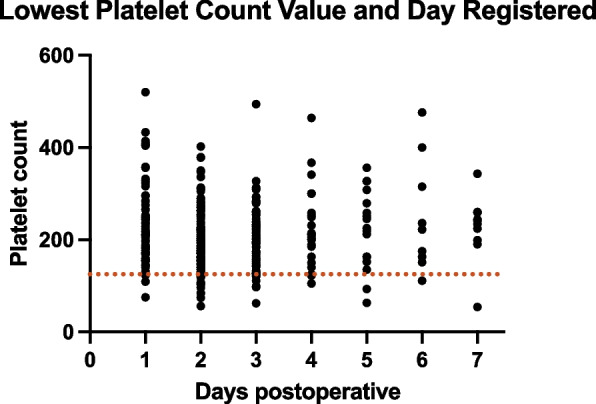
Lowest Platelet Count Value and Day Registered: The lowest platelet count values are recorded each day from preoperative day to postoperative days 1 through 7, highlighting the critical points where platelet counts reached their minimum during the postoperative period

For patients undergoing rTHA, the platelet count showed a significant decrease postoperatively, reaching its lowest value on day 2.5 ± 1.2, with a mean of 193.7 ± 73.1 × 10^9^/L (Figs. 1 and 2). The delta in platelet count from preoperative to the lowest postoperative value was 73.5 [− 229:368] × 10^9^/L (*P* < 0.001). The incidence of thrombocytopenia was significantly higher in the rTHA group at 28.7% (*P* < 0.001) (Table 1). The incidence of blood transfusions during hospitalization was significantly higher in the rTHA group at 51.7% (*P* < 0.001) (Table 1).

For the rTKA group, the platelet count showed a significant decrease postoperatively, reaching its lowest value on day 2.8 ± 1.7, with a mean of 231.1 ± 80.0 × 10^9^/L (Figs. 1 and 2). The delta in platelet count from preoperative to the lowest postoperative value was 46.0 [− 358:257] × 10^9^/L (*P* < 0.001). The incidence of thrombocytopenia in the rTKA group was 12.3% (*P* < 0.001) (Table 1). The incidence of blood transfusions during hospitalization in the rTKA group was 16.7% (*P* < 0.001) (Table 1). No clinically significant postoperative bleeding events, such as wound hematoma, hemarthrosis, or reoperation for bleeding, were observed in either group.

### White blood cell count and neutrophil count

**Fig. 3:**
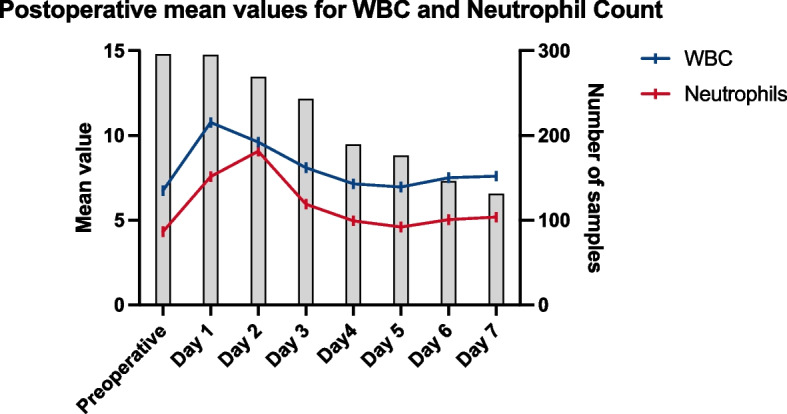
Postoperative mean values for white blood cell (WBC) and Neutrophil Count: Mean values for WBC counts and neutrophil counts from preoperative day to postoperative days 1 through 7. The figure shows changes over time, with separate plots for WBC and neutrophils, along with the number of samples taken each day

**Fig. 4:**
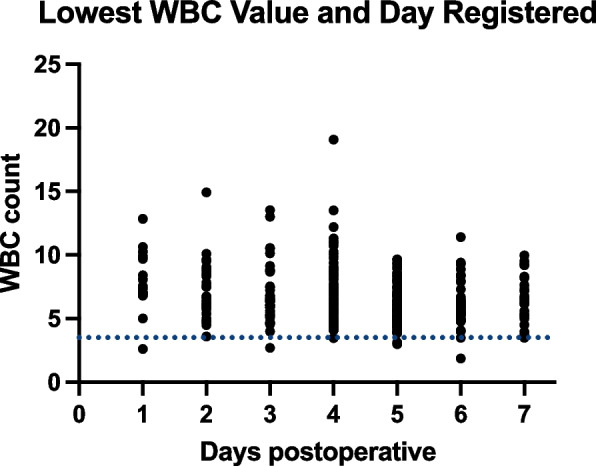
Lowest white blood cell (WBC) Value and Day Registered: The lowest WBC values are recorded each day from postoperative day 0 to day 7, indicating the days when the WBC count was at its lowest during the postoperative period

For the rTHA group, the WBC count showed an initial increase on postoperative day 1, followed by a gradual decrease, reaching its lowest value on day 4.4 ± 1.4, with a mean of 6.7 ± 2.2 × 10^9^/L (Figs. 3 and 4). The delta in WBC count from preoperative to the lowest postoperative value was 0.18 [− 12.1:6.6] × 10^9^/L. The incidence of leukopenia was 4.7% (*P* = 0.02) (Table 1).

**Fig. 5:**
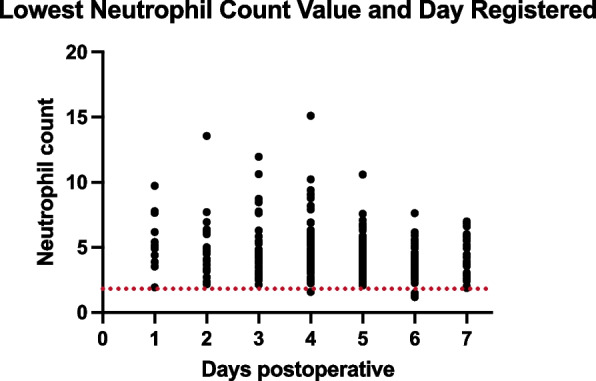
Lowest Neutrophil Count Value and Day Registered: The lowest neutrophil count values are recorded each day from postoperative day 0 to day 7, identifying the days with the minimum neutrophil counts during the postoperative recovery period

Similarly, the neutrophil count followed a comparable trend, with an initial increase on postoperative day 1 and a subsequent decline, reaching its nadir on day 4.6 ± 1.3, with a mean of 4.4 ± 1.9 × 10^9^/L (Figs. 1 and 5). The delta in neutrophil count from preoperative to the lowest postoperative value was − 0.15 [− 11.1:6.3] × 10^9^/L. The incidence of neutropenia was 2.6% (*P* = 0.02) (Table 1).

For the rTKA group, the WBC count showed an initial increase on postoperative day 1, followed by a gradual decrease, reaching its lowest value on day 4.2 ± 1.7, with a mean of 6.7 ± 2.0 × 10^9^/L (Figs. 3 and 4). The delta in WBC count from preoperative to the lowest postoperative value was 0 [− 0.9:6.4] × 10^9^/L. The incidence of leukopenia was 0.6% (*P* = 0.02) (Table 1).

Similarly, the neutrophil count followed a comparable trend, with an initial increase on postoperative day 1 and a subsequent decline, reaching its nadir on day 4.3 ± 1.7, with a mean of 4.5 ± 1.9 × 10^9^/L (Figs. 1 and 5). The delta in neutrophil count from preoperative to the lowest postoperative value was − 0.24 [− 10.1:7.2] × 10^9^/L. The incidence of neutropenia was 0% (*P* = 0.02) (Table 1).

### Multivariate linear regression

Undergoing rTHA (β 14.65; CI 8.05–21.25; *P* < 0.001) and low preoperative platelet levels (β 0.43; CI 0.37–0.48; *P* < 0.001) were independent risk factors for having a greater decrease in postoperative platelet levels. Antibiotic levels or duration were not significantly associated with the delta in platelet count (Table 2).

**Table 2:** Regression for delta in platelets

Variables	Estimate	*P* value	Lower 95%	Upper 95%
Age	0.7454384	0.0558	− 0.018433	1.5093093
Sex (0 = female; 1 = male) [0]	4.2781371	0.1591	− 1.686382	10.242656
Joint (1 = knee, 0 = hip) [0]	14.653597	<.0001	8.0528732	21.25432
CCI	− 0.982258	0.7627	− 7.379409	5.4148921
Platelets preop (× 10^9^/L)	0.4272851	<.0001	0.3699597	0.4846104
BMI	− 0.247579	0.7348	− 1.684922	1.1897642
IV antibiotic type vancomycin [0]	3.6087247	0.7313	− 17.05569	24.273142
IV antibiotic duration (d)	1.1920318	0.4704	− 2.054134	4.4381978
Topical antibiotic infusion type Antibiotic (0 = vancomycin; 1 = meropenem; 2 = imipenem; 3 = Vancomycin and imipenem; 4 = Vancomycin and meropenem) 2 [0]	1.2443464	0.7104	− 5.34622	7.8349131
Topical antibiotic duration (d)	0.0577787	0.9744	− 3.487929	3.6034867

*CCI* Charlson comorbidity index, *BMI* body mass index, *IV* intravenous, *preop* pre-operative

A univariate regression model showed that the delta in platelet levels was associated with an increased rate of postoperative transfusion (OR 1.008; CI 0.001–0.01; *P* = 0.017); however, with a minimal effect on this outcome (Table 2).

## Discussion

Single-stage exchange arthroplasty is gaining popularity for the treatment of PJI [10]. However, the impact of this treatment approach on hematologic parameters has not been thoroughly investigated. Our study aimed to examine the trends in postoperative blood cell counts among PJI patients treated with a combination of intravenous and intra-articular antibiotics. We found that single-stage exchange arthroplasty for PJI is associated with significant postoperative decreases in platelet count. The delta in platelet count was notably higher in patients undergoing hip revisions. These findings suggest the need for tailored perioperative management strategies to mitigate hematologic stress, including regular monitoring of blood parameters and early intervention to manage significant hematologic changes, ultimately improving patient outcomes.

To our knowledge, this is the first study to describe postoperative trends in commonly used Hematologic markers in single-stage PJI. In the present study, platelet count significantly decreased after surgery, reaching the nadir between the 2–3 postoperative day. Compared to rTKA, rTHA was associated with a greater decrease in platelet levels after surgery. These differences may be attributed to the more extensive surgical trauma and the greater blood loss, as indicated by higher transfusion rates, typically associated with hip revisions compared to knee revisions [23, 24]. One previous study by Intiso et al. described a high incidence of postoperative thrombocytosis 7 days after surgery, in patients undergoing primary total joint arthroplasty, among other aseptic hip and knee procedures [25]. The contrasting findings in this study can be attributed to the timing of the sample collection. PJI patients exhibit an acute fall in platelet levels on day 2, which normalizes at day 6–7 without reaching the cutoff for thrombocytosis. Similarly, the septic patient population can have a further decrease in platelet levels secondary to the infectious process [26]. In the aforementioned study, the high platelet levels were not associated with increased VTE rates, and there were no differences in demographics, which aligns with our findings.

Research indicates that significant drops in platelet count postoperatively can occur in patients treated with vancomycin, with incidences reported between 2.4% and 3.6% in comparative studies with linezolid [27, 28]. In our study, a sizeable part of the patients received intravenous and intra-articular vancomycin treatment. However, we did not observe any significant association between the type or duration of antibiotic administration and changes in postoperative platelet count. To our knowledge, there is Limited literature specifically addressing the Hematologic effects of intra-articular antibiotic infusion. Our findings suggest that this route may not contribute substantially to early postoperative thrombocytopenia. Nevertheless, early identification of significant drops in platelet count is crucial, as it can lead to severe and non-severe bleeding in 6% and 67% of cases, respectively [29]. Additionally, our data showed that greater reductions in platelet count were associated with an increased risk of postoperative transfusion, underscoring the clinical relevance of close hematologic monitoring. Further studies are needed to validate the hematologic safety of intra-articular antibiotic infusion in diverse patient populations and for longer-term follow-up.

The trends in WBC and neutrophil counts followed a similar pattern, with an initial postoperative increase indicative of an inflammatory response, followed by a gradual decline. The mean WBC and neutrophil counts reached their lowest values around postoperative day 4–5. Although the incidence of leukopenia and neutropenia was higher in the rTHA group compared to the rTKA group, the differences were not statistically significant.

These findings align with previous studies that have noted an early postoperative increase in inflammatory blood cells due to surgical trauma [11]. The subsequent decline in WBC and neutrophil counts may reflect the resolution of the acute inflammatory response. In orthopedic infection cases, Rao et al. reported a significant mean decrease in the neutrophil count by 41% from baseline within the vancomycin group, highlighting the critical need for vigilant monitoring of blood counts in patients undergoing prolonged vancomycin therapy [15]. However, our results did not show such a severe decrease. Instead, the changes we observed were less dramatic, underscoring the importance of regular monitoring of blood counts. Our data indicated that the type and duration of antibiotic therapy (including vancomycin and/or carbapenem) did not significantly impact the changes in WBC and neutrophil counts. While the number of patients who reached the cutoff for leukopenia and neutropenia was negligible, monitoring of these markers could be beneficial to analyze isolated peaks and rule out early postoperative infection.

Our findings suggest that on postoperative day 2–3 after single-stage surgery for PJI, platelets are expected to decrease by an average of 73.1 × 10^9^/L in rTHA and 46 × 10^9^/L in rTKA. Future research should aim to establish standardized protocols for the monitoring and management of hematologic parameters in this patient population. Additionally, studies exploring the combined impact of surgical trauma, infection, and prolonged antibiotic therapy on hematologic changes are warranted. Understanding these mechanisms could lead to the development of targeted interventions that minimize hematologic complications, improving postoperative outcomes.

Intra-articular antibiotic infusion was employed postoperatively in our study as a targeted strategy to sustain high local antibiotic concentrations within the joint cavity during the early postoperative phase. This method is particularly relevant in the context of biofilm-associated infections, where bacteria embedded within biofilms may require antibiotic concentrations several hundred to thousands of times higher than those needed to eliminate planktonic organisms [30]. By administering scheduled intra-articular doses, consistent antibiotic exposure can be maintained locally while limiting systemic toxicity [31–33]. Moreover, this technique allows for timely adjustments in antibiotic selection based on intraoperative culture results, providing therapeutic flexibility in complex scenarios such as culture-negative infections, polymicrobial cases, or revision procedures following prior treatment failures, settings in which favorable mid-term outcomes were reported in our previous studies [4, 6–8]. The protocol was well tolerated and may serve as a valuable adjunct in infection control following single-stage revision arthroplasty [34, 35].

This study should be interpreted in the context of several limitations. The retrospective design may introduce bias due to reliance on historical medical records, which can affect the completeness of the data. The single-center setting may limit the generalizability of the findings to other populations and healthcare settings, as practices and patient demographics can vary significantly. Intraoperative variables such as operative time, estimated blood loss, and the extent of surgical invasiveness, including pelvic reconstruction in selected hip revision cases, were not systematically documented and therefore not included in the analysis. These factors may influence postoperative hematologic responses by affecting intraoperative blood loss, tissue injury severity, and systemic inflammation, potentially altering platelet consumption patterns or transfusion requirements. The study did not include dynamic monitoring of hemoglobin (Hb) levels. Including Hb dynamics would offer a more complete picture of the Hematologic changes occurring postoperatively. Additionally, our study only monitored Hematologic parameters for the first 1–7 days postoperatively, which Limits our ability to analyze changes beyond the first week. Given that antibiotic therapy often extends beyond this timeframe, we cannot directly assess Hematologic changes occurring after the initial 7 days. Moreover, we did not include outcomes related to PJI treatment, such as infection recurrence and other complications, in our analysis. Furthermore, the retrospective study did not further analyze the impact of different anticoagulation regimens and the use of tranexamic acid on platelet counts and transfusion requirements. It should also be noted that intra-articular antibiotic infusion, although commonly used in our institution, is not yet a universally adopted practice. While no significant associations with platelet decline were observed in our cohort, the potential hematologic effects of this technique remain insufficiently studied in the broader literature and require further validation in future prospective investigations. Addressing these limitations in future research by incorporating dynamic Hb monitoring, standardized assessment timings, and longer follow-up periods, as well as including clinical outcomes related to PJI treatment, would enhance the clinical relevance of the findings and help develop more effective management strategies.

## Conclusions

Our study shows that platelet count significantly decreases during the 2–3 postoperative days after single-stage PJI revision surgery, particularly in rTHA patients. However, no clinically significant bleeding events were observed, indicating that this Hematologic change did not translate into increased bleeding risk. Additionally, intravenous or intra-articular antibiotic therapy seems not to affect postoperative platelet, WBC, or neutrophil count. Future efforts should focus on continuous monitoring of these markers for intervals beyond 7 days and evaluating the impact of these fluctuations on clinical outcomes.

## Data Availability

No datasets were generated or analysed during the current study.

## Abbreviations

PJI: Periprosthetic joint infection
WBC: White blood cell
rTHA: Revision total hip arthroplasty
rTKA: Revision total knee arthroplasty
ESR: Erythrocyte sedimentation rate
CRP: C-reactive protein
VTE: Venous thromboembolism
CCI: Charlson comorbidity index
SD: Standard deviation
IQR: Interquartile range
Hb: Hemoglobin
